# The Atomic, Molecular and Optical Science instrument at the Linac Coherent Light Source

**DOI:** 10.1107/S1600577515004646

**Published:** 2015-04-17

**Authors:** Ken R. Ferguson, Maximilian Bucher, John D. Bozek, Sebastian Carron, Jean-Charles Castagna, Ryan Coffee, G. Ivan Curiel, Michael Holmes, Jacek Krzywinski, Marc Messerschmidt, Michael Minitti, Ankush Mitra, Stefan Moeller, Peter Noonan, Timur Osipov, Sebastian Schorb, Michele Swiggers, Alexander Wallace, Jing Yin, Christoph Bostedt

**Affiliations:** a Linac Coherent Light Source, SLAC National Accelerator Laboratory, 2575 Sand Hill Road, Menlo Park, CA 94025, USA; b Department of Applied Physics, Stanford University, 348 Via Pueblo, Stanford, CA 94305, USA; c Pulse Institute, Stanford University and SLAC National Accelerator Laboratory, 2575 Sand Hill Road, Menlo Park, CA 94025, USA

**Keywords:** FEL, X-ray, ultrafast, spectroscopy, imaging

## Abstract

The Atomic, Molecular and Optical Science (AMO) instrument at the Linac Coherent Light Source (LCLS) provides a tight soft X-ray focus into one of three experimental endstations. The flexible instrument design is optimized for studying a wide variety of phenomena requiring peak intensity. There is a suite of spectrometers and two photon area detectors available. An optional mirror-based split-and-delay unit can be used for X-ray pump–probe experiments. Recent scientific highlights illustrate the imaging, time-resolved spectroscopy and high-power density capabilities of the AMO instrument.

## Abstract 2

A description of the Atomic, Molecular and Optical Sciences (AMO) instrument at the Linac Coherent Light Source is presented. Recent scientific highlights illustrate the imaging, time-resolved spectroscopy and high-power density capabilities of the AMO instrument.

## 1. Introduction   

The Atomic, Molecular and Optical Science (AMO) instrument is tailored to provide a tight focus with the maximum possible photon flux for experiments requiring high peak intensity in the soft X-ray spectral regime at the Linac Coherent Light Source. The AMO hutch is located in the Near Experimental Hall (NEH), approximately 140 m downstream of the undulators. The instrument consists of a pair of Kirkpatrick–Baez (KB)-mirror focusing optics, beam diagnostics, an optional split-and-delay unit, and three diverse endstations with various sample delivery capabilities. A femtosecond optical laser system is available for optical–X-ray pump–probe experiments.

Typical experiments at the AMO instrument range from high-intensity X-ray spectroscopy (Young *et al.*, 2010 ▶; Berrah *et al.*, 2011 ▶; Doumy *et al.*, 2011 ▶; Rudek *et al.*, 2012 ▶) to time-resolved and pump–probe experiments (Cryan *et al.*, 2010 ▶; Meyer *et al.*, 2012 ▶; Schorb *et al.*, 2012*a* ▶; McFarland *et al.*, 2014 ▶) as well as coherent diffractive imaging of biological objects (Seibert *et al.*, 2011 ▶; Kassemeyer *et al.*, 2012 ▶), aerosols (Loh *et al.*, 2012 ▶), clusters (Gorkhover *et al.*, 2012 ▶) and gas-phase laser-aligned molecules (Küpper *et al.*, 2014 ▶).

In the following we will give an overview of the AMO instrument including the various endstations, X-ray optics, optical laser systems and available detectors. We conclude with three research highlights showcasing the instrument capabilities.

## 2. Instrument overview   

**Figure 1:**
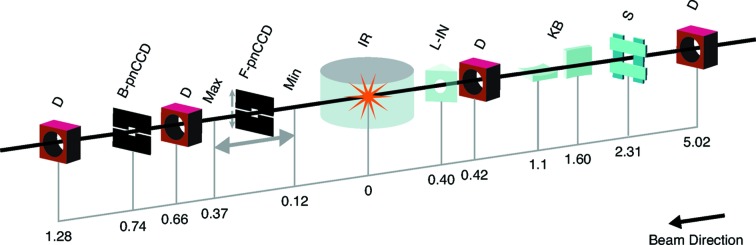
Overview of the AMO instrument layout with the LAMP endstation. Distances are indicated in meters from the interaction region (IR). The X-ray beam enters the hutch and can first be visualized on a diagnostic (D) screen 5 m upstream of the IR. The beam passes through the aperture slits (S) and is focused by the KB optics (KB). An optical laser in-coupling (L-IN) mirror is located 0.4 m upstream of the IR. The front pnCCD (F-pnCCD) and back pnCCD (B-pnCCD) is located downstream of the IR. A set of three manipulators between the pnCCDs is usable as a beam-position diagnostic, an optical absorption filter or a protective B_4_C beamstop. Optional diagnostics are located after the back pnCCD, 1.28 m downstream of the IR. The X-ray split and delay unit can be inserted between the KB system and the L-IN which shifts everything behind the KB system 1 m further downstream. The AMO instrument is located approximately 140 m downstream of the undulators.

The AMO instrument provides three different endstations for a wide variety of applications (Bostedt *et al.*, 2013 ▶). LAMP, the latest endstation addition, is a versatile setup for coherent diffractive imaging and spectroscopy applications. This endstation consists of three independent sections. The naming convention for the sections follows its predecessor, the CAMP endstation (Strüder *et al.*, 2010 ▶), *i.e.* the interaction chamber (C1), the front pnCCD (see §2.3) holding chamber (C2-1), and the rear pnCCD holding chamber (C2-2). C1 is a flexible interaction chamber allowing easy integration of the available spectrometers and sample sources (*cf.* Table 1 ▶). Further, C1 provides an in-vacuum laser breadboard and three sets of piezo-motor stages for mounting, *e.g.* samples, optics or apertures. The front pnCCD can be moved along the X-ray beam from 121 mm to 371 mm downstream of the interaction region, which allows scattering angles on the front pnCCD up to 55° and 25°, respectively. A set of three manipulators in C2-1 is usable as beam position diagnostics, optical absorption filters or a protective B_4_C beam stop in front of the rear pnCCD. The rear pnCCD is located 737 mm downstream of the interaction point and can detect scattering angles of up to 4°. A large gate-valve is located between the C1 and C2 chambers, separating the interaction region and cooled pnCCD detectors, thus allowing rapid intervention in C1 during beam time. The entire system, depicted in Fig. 1 ▶, is designed to handle high gas loads and ultra-high vacuum (UHV) needs.

The second AMO experimental endstation, the High-Field Physics (HFP) system, is optimized for high-resolution ion and electron spectroscopy. The HFP endstation is fitted with a double-layer µ-metal shielding to attenuate extraneous magnetic fields in the interaction region. The HFP endstation consists of five electron time-of-flight spectrometers and one ion time-of-flight spectrometer. Of the five electron spectrometers three are located in the plane perpendicular to the X-ray beam at 90°, 0° and the ‘magic angle’ 54.7° with respect to the polarization axis. The fourth is oriented at the ‘magic angle’ containing the beam propagation and polarization axis, and the last one is oriented at the ‘magic angle’ containing the X-ray beam direction and the axis normal to the polarization. The ion spectrometer has holes in the repeller plate for electron trajectories, so that both the electron spectrometers and the ion spectrometer can be mounted for the same experiment.

A third experimental endstation, the Diagnostics (DIA) endstation, was designed to perform shot-by-shot analysis of the X-ray pulse spectral profile. The endstation is complete with large in-vacuum breadboards and optical elements. The chamber’s large diameter allows for optical–X-ray pump–probe experiments with a large footprint.

All device components are compatible with each end­station, unless otherwise specified. The soft X-ray capabilities of LCLS and AMO instrument details are summarized in Table 1 ▶. Further details about the endstations are given by Bostedt *et al.* (2013 ▶).

### 2.1. X-ray optics   

The X-ray beam is deflected into the AMO hutch by three mirrors. They are coated with B_4_C and exhibit a 14 mrad angle of incidence. The KB-mirror system is the essential X-ray focusing tool in AMO and comprises two 400 mm-long silicon substrates with a 50 nm B_4_C reflective coating. Both mirrors are bendable in a plane-elliptical geometry which enables the foci to be varied dynamically along the instrument from the optimal focal plane to infinity. The angle of incidence for both mirrors is 13.85 mrad and the designed focal length of the unit is 1600 mm and 1100 mm for the horizontal and vertical focusing mirrors, respectively. An adjustable aperture system limits the illumination on the mirrors for stray light impairment.

An all-optical soft X-ray split-and-delay (XRSD) unit is optionally available to wavefront split an incoming X-ray pulse into two time-separated identical pulses. The device operates by using two silicon mirrors positioned along the X-ray beam path. The first mirror cuts a portion of the beam and deflects it at a very shallow angle towards the interaction region. The second mirror is positioned downstream of the first and deflects the remaining portion of the beam along a slightly larger angle towards the interaction region. The silicon mirrors operate at angles less than 13.5 mrad and fit into an approximately 1 m space along the instrument. The XRSD unit can provide two X-ray pulses separated by up to 200 fs, with femtosecond time resolution, and operates over the soft X-ray range from 280 to 1800 eV.

### 2.2. Optical laser capabilities   

All AMO endstations provide the capability to use optical lasers in co-linear geometry with the X-ray beam for optical–X-ray pump–probe experiments. Various X-ray–optical cross correlators are available to measure the timing jitter between the X-ray and optical lasers (Bionta *et al.*, 2014 ▶; Schorb *et al.*, 2012*b* ▶; Hartmann *et al.*, 2014 ▶).

Core laser systems at the LCLS consist of an ultrashort-pulse Ti:sapphire oscillator synchronized to the FEL. The oscillator seeds a commercially available chirped pulse amplifier producing 4 mJ at 40 fs. An additional home-built four-pass amplifier can boost the pulse energy to over 30 mJ. Wavelength conversion inside the hutch can cover a broad spectral range from 200 nm to 150 µm (2 THz). A more in-depth description of the optical laser capabilities at LCLS is given by Minitti *et al.* (2015 ▶).

### 2.3. Detectors   

A suite of charged-particle spectrometers is available at the AMO instrument. A high-resolution double-sided electron-ion coincidence velocity map imaging (VMI) spectrometer specifically designed for use in the LAMP endstation detects ions and/or electrons while providing a clear line of sight from the interaction region to the pnCCD detectors. The ion side of the spectrometer can detect kinetic energies of up to 50 eV with time-of-flight resolution of 100 ps. The position-sensitive 120 mm quad delay line detector yields a resolution of 250 µm. The standard configuration electron side of the spectrometer contains a phosphor screen detector with an energy resolution 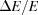 up to 1/100, with the ability to measure up to 150 eV electrons. An optional hex anode is available to replace the phosphor screen. Other spectrometers available to measure charge states, kinetic energies and momenta of ions are an integrating spectrometer (Bozek, 2009 ▶), a VMI spectrometer (Eppink & Parker, 1997 ▶) and a reaction microscope ion spectrometer (Dorner *et al.*, 2000 ▶).

The LAMP endstation is equipped with two single-photon-counting pnCCDs (Strüder *et al.*, 2010 ▶). Each detector consists of two large-area (78 mm × 37 mm) pnCCD sensors (75 µm × 75 µm pixel size). The pnCCDs collect scattered or fluorescence photons with high quantum efficiency and an energy resolution of 40 to 200 eV between 50 eV and 25 keV at a frame read-out rate of up to 250 Hz. The first pnCCD is mounted on a moving stage to produce a variable size gap between the two sensor halves. The second CCD is mounted on a fixed frame with a 3.8 mm × 3.8 mm square hole in the center for the direct FEL beam. Each detector can be operated in high-resolution-imaging or spectroscopy mode.

## 3. Highlights   

The AMO instrument has been used in a wide range of scientific investigations ranging from AMO to materials and high-energy-density sciences as well as single-shot coherent imaging applications (Bostedt *et al.*, 2013 ▶). The following three examples illustrate three different capabilities of the instrument.

### 3.1. Coherent diffractive imaging of rotating superfluid nanodroplets   

**Figure 2:**
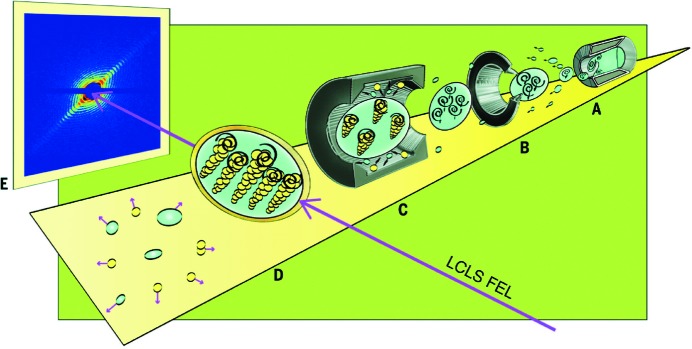
The experimental setup for imaging rotating superfluid helium nanodroplets. (A) Rotating droplets are formed by expanding helium fluid into vacuum. (B) The nanodroplets become superfluid after evaporative cooling. (C) Droplets are optionally doped with Xe atoms in a gas cell. (D) and (E) X-ray diffraction images from single nanodroplets are recorded with the pnCCD. Figure reprinted with permission from Gomez *et al.* (2014 ▶).

Superfluid helium is a quantum mechanical state that extends over macroscopic length scales, much like Bose–Einstein condensates and superconductors. In a recent coherent diffractive imaging experiment superfluid rotating helium nanodroplets are placed into the X-ray focus and their scattering patterns are recorded with pnCCD detectors (Gomez *et al.*, 2014 ▶). The superfluid ^4^He droplets are formed *via* expansion of high-purity helium through a 5 µm-diameter nozzle at a temperature of 5 K and evaporative cooling lowers the droplet temperature below the superfluid transition at 2.17 K. Optionally, the helium nanodroplets could be doped with xenon atoms in a pickup cell. The xenon atoms exhibit a much higher scattering cross section than the helium atoms at X-ray energies and thus can act as X-ray contrast agent. A schematic of the experiment is depicted in Fig. 2 ▶.

Some of the diffraction images from pristine nanodroplets exhibit sharp streaks as shown in Fig. 2(E) ▶. Such diffraction images indicate that the nanodroplets are extremely flat, or ‘wheel-shaped’ with two almost parallel surfaces. From the extreme shape distortions it can be concluded that the droplets spin with rotational velocities beyond classical stability limits. The data show that superfluid nanodroplets behave very differently from their classical counterparts.

Any rotational motion in a superfluid is manifested in quantum vortices. In a second step of the experiment xenon-doped superfluid droplets are investigated. The xenon atoms cluster along vortex cores. This way the quantum vortices can be directly imaged. The diffraction data show Bragg spots on top of characteristic helium droplet ring patterns.

The quantum vortices can be directly imaged *via* the xenon atoms that dope the helium nanodroplet. The Bragg peak separations correspond to regularly spaced xenon structures, which indicate that the helium droplets contain a regularly spaced vortex lattice. The observed vortex densities are orders of magnitude larger than in bulk superfluid helium.

This experiment utilizes the imaging capabilities of the AMO instrument to unambiguously demonstrate a quantum mechanical state of motion for an entire helium nanodroplet.

### 3.2. Charge transfer upon X-ray photoabsorption   

**Figure 3:**
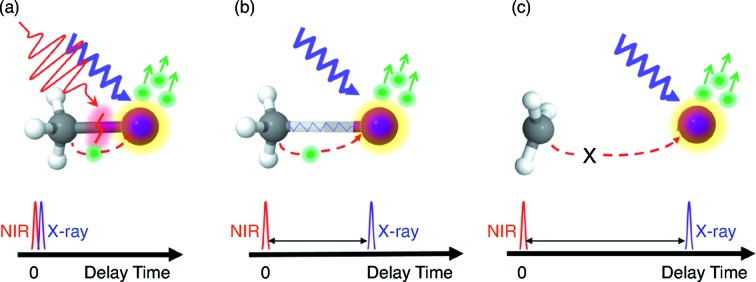
The experimental concept for investigating ultrafast charge transfer processes in CH_3_I. An 800 nm NIR pulse dissociates the molecule. The X-ray pulse arrives after a certain delay, and thus molecular separation, and creates charge predominantly at the I atom. At short delays (*a*), the charge is shared between the two fragments. In the intermediate regime (*b*), the charge distribution between the fragments depends on the interatomic distance. At long delays (*c*), the interatomic distance is too large and charge transfer becomes negligible. Figure reprinted with permission from Erk *et al.* (2014 ▶).

Charge transfer processes drive many important transformations in physics, chemistry and biology. Determining the spatial localization of charge at a given time remains a key difficulty. A recent study by Erk *et al.* (2014 ▶) ▶ directly mapped charge transfer dynamics upon inner-shell ionization of iodomethane (CH_3_I) at the AMO instrument. As Fig. 3 ▶ shows, the CH_3_I molecule is first dissociated with a near-infrared (NIR) laser and then ionized with an intense X-ray laser pulse. The internuclear separations between the CH_3_ and atomic I fragments are defined by the time delay between the dissociating NIR and ionizing X-ray pulses. Inner-shell photoionization and Auger decay induce a positive charge that is initially strongly localized over the iodine atom. The charge then spreads over the entire molecule *via* separate processes depending on the internuclear separation at the time of ionization.

Measuring charge state and kinetic energy distributions of the fragment ions as a function of NIR–X-ray delay allows for a detailed analysis of charge redistribution between the CH_3_ and I fragments based on their internuclear separation. For delays within 100 fs the interatomic distances are so small that at least one valence electron always leaves the methyl group fragment. Between 100 and 300 fs the electrons are fairly localized, and the electron-transfer probability is dependent on the interatomic separation. After 300 fs the distance between the methyl group and iodine fragments becomes too large and charge transfer between iodine and carbon becomes highly improbable. The data can be well described by a classical ‘over-the-barrier’ charge transfer model; at a critical separation the height of the classical potential barrier between the two bodies becomes larger than the binding energy of the valence electrons.

This example provides a technique for spatio-temporal imaging of charge transfer dynamics, and features the time-resolved capabilities of the AMO instrument.

### 3.3. Stimulated processes: from X-ray lasing to inelastic Raman scattering   

The unprecedented intensities from X-ray free-electron lasers (XFELs) opens the door for stimulated processes in the X-ray spectral regime. In a first proof-of-principle experiment at the AMO instrument the intense LCLS pulses have been used to drive an atomic inner-shell laser in a dense gas of neon (Rohringer *et al.*, 2012 ▶). The inner-shell vacancies created in the neon 1*s* level upon X-ray absorption decay dominantly *via* Auger processes. However, there is a small probability for a spontaneous radiative decay, emitting a photon with an energy of 849 eV. These photons can be exponentially amplified along the plasma channel in the dense gas created by the free-electron laser pulse. For detection of the lasing signal the outgoing beams are dispersed with a grating spectrometer where the atomic lasing line can be distinctly distinguished.

**Figure 4:**
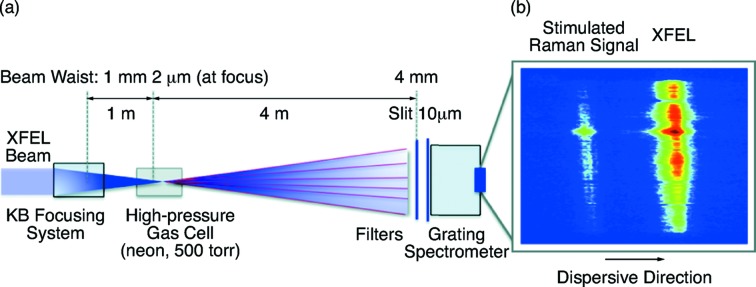
Stimulated Raman scattering setup at the AMO instrument. In (*a*) the XFEL beam is focused by the KB optics system into a high-pressure gas cell filled with neon. A grating spectrometer is used to separate the FEL and Raman signal. The Raman signal and FEL pulse in (*b*) are separated by approximately 20 eV. Figure reprinted with permission from Weninger *et al.* (2013 ▶).

In a conceptually similar experiment a stimulated X-ray Raman signal has been observed (Weninger *et al.*, 2013 ▶). Here, the photon energy is tuned below the neon *K*-edge ionization threshold. The photon energy is tuned to around 870 eV (*K*-edge of neon) and a stimulated Raman signal is observed at 850 eV. The experimental scheme is shown in Fig. 4 ▶. The resonant excitation by the intense X-ray pulses outruns the Auger decay and thus create a population inversion. A radiative decay of electrons into the 1*s*–2*p* states results in exponential amplification of the Raman signal. The stimulated Raman signal is separated from the incoming photon energy by approximately 20 eV. It can be clearly identified on the grating spectrometer located about 4 m behind the gas cell along the beam axis.

This study uses the high X-ray power density available in the AMO instrument to demonstrate stimulated emission processes, opening the door for non-linear spectroscopy approaches.

## 4. Conclusion   

The LCLS produces high-flux few-femtosecond X-ray pulses, yielding unprecedented X-ray intensities. The AMO instrument takes advantage of the pulse properties to perform high-power soft X-ray experiments in a wide spectrum of scientific domains. The instrument provides users with a variety of endstations, spectrometers and other components for the utmost flexibility in experimental layouts and signal detection schemes. More details about the AMO instrument can be found on the following website: http://lcls.slac.stanford.edu/amo.

## 5. Facility access   

LCLS instruments are open to academia, industry, government agencies and research institutes worldwide for scientific investigations. There are two calls for proposals per year and an external peer-review committee evaluates proposals based on scientific merit and instrument suitability. Access is without charge for users who intend to publish their results. Prospective users are encouraged to contact instrument staff members to learn more about the science and capabilities of the facility, and opportunities for collaboration.

## Figures and Tables

**Table 1:** X-ray parameters and capabilities of the AMO instrument

Instrument name	AMO
Mirrors, incidence angle	3 B_4_C on Si, 14mrad
Monochromaticity ( 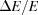 )†	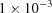 (SASE), 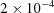 (seeding)
Energy range (eV)	2802000 ▶
Unfocused beam size (m)	2700 at 700eV
Focused beam size (m)	1.5
Focusing optics	Bendable KB (B_4_C on Si pair)
Flux (photonspulse^1^)	Up to 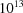 ‡
Pulse length (fs)	5200
Repetition rate (Hz)	120, 60, 30, 10, 5, 1, on demand
Optical laser pulse energy (mJ)	20 (800nm), 45 (400nm), 1 (266nm)
Optical laser pulse width (fs)	10150
Sample delivery	Even-Lavie valve, Parker valve, XYZ stage
Standard chambers	LAMP, HFP, DIA
Standard photon detectors	Two large-area pnCCDs
Standard spectrometers	Ion/electron VMI/reaction microscope
	5 electron TOF, 1 ion TOF
	Ion momentum TOF, VMI

† Typical single-shot value.

‡ Excluding beamline and instrument transmission.
